# Supplementary material for: Exploring the association between sleep quality, internet addiction, and related factors among adolescents in Dakshinkali Municipality, Nepal
Source: PLoS One. 2025 Jan 22;20(1):e0317747. doi: 10.1371/journal.pone.0317747 (PMC11753655; doi:10.1371/journal.pone.0317747)
Supplement: Attachment — Submitted filename: Reviewers comments 20240509.docx [file pone.0317747.s002.docx]

The reviewer’s comments for the manuscript:

**Title:** The specific research objectives are not evident from the title. Please revise accordingly.

**Methodology:**

(1) The assessment tools should be detailed separately under the 'Measurements' section.
(2) Please provide a description of the forward and backward translation process to ensure the reliability and validity of the tools.
(3) The regression analysis section should clarify the dependent and independent variables.
(4) Adjust the analysis method in Tables 2 and 3 as follows: If the original data are continuous variables, use t-tests or ANOVA to analyze the differences between groups. Additionally, the authors do not clarify the cut-off points for each variable and their rationales, such as sleep quality, severity of internet addiction, and the quality of family and teacher-student relationships.
(5) The relationships between sleep and internet addiction may need to be explored using Structural Equation Modeling (SEM).
(6) Important variables related to adolescent sleep issues were omitted during data collection, including emotional factors (e.g., anxiety, depression), physical health, daily routines, academic load, and extracurricular activities. Thus, the explanatory power of the study results is limited. It is recommended to include these variables.

Overall Judgment: Recommend a Major Revision.
