# Supplementary material for: Exploring the association between sleep quality, internet addiction, and related factors among adolescents in Dakshinkali Municipality, Nepal
Source: PLoS One. 2025 Jan 22;20(1):e0317747. doi: 10.1371/journal.pone.0317747 (PMC11753655; doi:10.1371/journal.pone.0317747)
Supplement: Attachment — Submitted filename: Response to Reviewers.docx [file pone.0317747.s003.docx]

The reviewer’s comments for the manuscript:

**Title:** The specific research objectives are not evident from the title. Please revise accordingly.

- Thank you for your valuable feedback. Considering your comment, we have revised our article title as “**Exploring the Association between Sleep Quality, Internet Addiction, and Related Factors among Adolescents in Dakshinkali Municipality, Nepal”** making it more reflective to our research objectives.

**Methodology:**

(1) The assessment tools should be detailed separately under the 'Measurements' section.

- Thank you for your suggestion regarding the assessment tools. We have reorganized the methodology section and created a separate "Measurements" sub-section. This section now provides detailed information about PSQI and IAT along with the cutoff we used to categorize the attributes.

(2) Please provide a description of the forward and backward translation process to ensure the reliability and validity of the tools.

- Thank you for notifying this missing information. We have tried to add some details on process adopted for translation and back-translation of the tool.

(3) The regression analysis section should clarify the dependent and independent variables.

- Thank you for suggestion, we have tried to make it more clear in the regression analysis part within the result section.

(4) Adjust the analysis method in Tables 2 and 3 as follows: If the original data are continuous variables, use t-tests or ANOVA to analyze the differences between groups. Additionally, the authors do not clarify the cut-off points for each variable and their rationales, such as sleep quality, severity of internet addiction, and the quality of family and teacher-student relationships.

- Thank you for your feedback. We employed clear cutoffs to categorize sleep quality and internet addiction severity (which me have tried to clear out in methodology section.) This approach allows for better comparability with national and international studies that have adopted similar categorization methods. Additionally, using chi-square allows us to explore the differences in variables across these categories.
- In regards to cut-off points for sleep quality, and internet addiction, we used cutoff suggested by original tool and previous studies.
- Cutoff we used for Sleep quality
  - PSQI is a four-point Likert scale ranging from 0 to 3 for each 19 items with the overall PSQI score ranging from 0 to 21. The lower score represents better sleep quality and the cut-off value of ≤5 can be used to denote good sleep quality
    - <https://www.sciencedirect.com/science/article/abs/pii/0165178189900474?via%3Dihub>
    - <https://www.sciencedirect.com/science/article/abs/pii/S138994570600551X?via%3Dihub>
- Cutoff we used for Internet Addiction
  - Scores obtained from the IAT are grouped into four categories: normal (0–30), mild IA (31–49), moderate IA (50–79), and severe IA (80–100).
- <https://www.iitk.ac.in/counsel/resources/IATManual.pdf> COPYRIGHT © by Dr. Kimberly S. Young All rights reserved. Center for Internet Addiction Recovery, P.O. Box 632, Bradford, PA 16701
- Internet addiction validation study in Indonesian adolescents (<https://www.ncbi.nlm.nih.gov/pmc/articles/PMC7861384/#:~:text=The%20total%20scores%20of%20IAT,)%20%5B5%2C19%5D>).
- Kumar N, Kumar A, Mahto SK, et al. Prevalence of excessive internet use and its correlation with associated psychopathology in 11th and 12th grade students. General Psychiatry 2019;32:e100001. doi:10.1136/ gpsych-2018-100001
- Subjective sleep quality, Sleep duration, Sleep efficiency, Sleep disturbance, use of sleep medications and Day time dysfunction are derived from 19 self-reported items of PSQI using the suggested procedure
  - <https://www.goodmedicine.org.uk/files/assessment,%20pittsburgh%20psqi.pdf> (Buysse, DJ, Reynolds CF, Monk TH, Berman SR, Kupfer DJ: The Pittsburgh Sleep Quality Index (PSQI): A new instrument for psychiatric research and practice. Psychiatry Research 28:193-213, 1989)
  - We have added a sentence in measurement sub-section under Data collection section, to make it more clear for the readers. Thank you for mentioning this missing detail
- In regards to Participants relation with their Friends, Family and Teachers, rather than looking at quality of family and teacher-student relationships we had simply asked participant to rate their Perceived nature of relation with them. We have tried to make it more clear in the manuscript Data collection section.

(5) The relationships between sleep and internet addiction may need to be explored using Structural Equation Modeling (SEM).

- Thank you for this suggestion, we highly appreciate it. Structural Equation Modeling (SEM) can be a powerful tool for examining complex relationships. However, in our cross-sectional study design, we are not looking to establish causal relationships. Our study focused on assessing associations between sleep quality and internet addiction so we went for Regression analysis. We agree that future research employing longitudinal designs and SEM to elucidate causal pathways between these variables would be beneficial. Considering your feedback, we have acknowledged this limitation in our manuscript.

(6) Important variables related to adolescent sleep issues were omitted during data collection, including emotional factors (e.g., anxiety, depression), physical health, daily routines, academic load, and extracurricular activities. Thus, the explanatory power of the study results is limited. It is recommended to include these variables.

- Thank you for this valuable feedback. We acknowledge that including additional variables related to adolescent sleep issues, such as emotional factors (anxiety, depression), physical health, daily routines, academic load, and extracurricular activities, could provide a more comprehensive understanding of the factors influencing sleep quality among adolescents with internet addiction. We recognize the limitations of our current study and the potential benefits of including these variables in future research. We have incorporated this limitation in the revised manuscript.
