# Supplementary material for: Exploring the association between sleep quality, internet addiction, and related factors among adolescents in Dakshinkali Municipality, Nepal
Source: PLoS One. 2025 Jan 22;20(1):e0317747. doi: 10.1371/journal.pone.0317747 (PMC11753655; doi:10.1371/journal.pone.0317747)
Supplement: Attachment — Submitted filename: Reviewers response.docx [file pone.0317747.s004.docx]

We would like to thank all the reviewers for their valuable time and comments provided for improvement of our manuscript. We hereby share with you our response to all the comments provided. Thank you

Reviewer #2:

The reference numbers in the text need to be reviewed as they are following after full stops. The fonts seem to be different as well.

- Thank you for notifying us about the placement of references and font consistency. We have revised the manuscript to place all reference numbers before the full stops. Additionally, we have ensured uniformity in font usage across the manuscript, adhering to Times New Roman throughout.

Reviewer #3:

 sample size: the author needs to justify the choice of 30% non-response rate
Authors can elaborate and define the independent and dependent variables in the statistical analysis section

- Thank you for your valuable time and suggestions. In regards to 30% non-response rate, it is the part of our initial methodology, we used the maximum limit of non-response rate that is usually practiced to optimize the sample size. We wanted keep our sample as optimum as possible as it was unclear how many participants will be able to provide their complete response in self-administered questionnaire. As mentioned in our result section our achieved response rate is 87% as among 280 participants approached, 243 provided complete response (yielding a non-response rate of 13%). This way we were able to secure the minimum required sample of 215.
- Thank you for this suggestion. We have revised the measurements section to explicitly define the independent variables and dependent variables.

Ethical consideration: The author should make clear that participant can withdraw from the study at any point of time without having any repercussions on them or on their performance in the academic institution they belong to.

- Thank you for your valuable suggestion. The right to withdraw at any point in the data collection was respected, which is the reason for getting complete response from 243 while 280 were approached. We have added the missing information about right to withdraw in ethical statement section. Thank you.

Reviewer #4:

In the current manuscript titled "Exploring the Association between Sleep Quality, Internet Addiction, and Related Factors among Adolescents in Dakshinkali Municipality, Nepal", Acharya et al have explored the prevalence and contributing factors of poor sleep quality and internet addiction among adolescents in a Nepali community of Dakshinkali Municipality. Gathering data from 243 school adolescents using scaling tests to measure sleep quality and internet addiction, the authors have reported that a quarter of adolescents in the study were reported to experience poor sleep quality and nearly half screened positive for potential internet addiction. While the manuscript offers a lot of information that will be useful to the sleep, psychological, immunology and behavioral communities, there are following comments that need to be addressed to further improve the quality of this article:

- Thank you for your valuable time and feedback provided to improve our manuscript.

1. In the "Sampling Size and Sampling Procedure", please describe briefly what was the non-response rate of 30% due to and what could be done to further improve this rating in the future studies

- The right to withdraw at any point in the data collection was respected which is the reason for getting complete response from 87% of approached participants. The non-response of 30% was the part of our methodology section as we were unsure how many participants will provide complete response and how many participants under 17 years will bring signed parental consent. So, to be on the safe side and meet the minimum sample of 215, we adjusted 30% non-response during the calculation of our sample size. However, the true non-response during the data collection of this study was 13%. We feel that we need to respect the voluntary participation of the participants, and at the same time not meeting the minimum required sample could lead to misrepresentation of the findings, so we adjusted the non-response to optimize the sample so that minimum sample could be secured. If there are no time constrains, then non-response rates can also be minimized by incorporating enhanced participant engagement strategies, such as sending reminders, follow up with the participants reporting missing information and parental involvement workshops, before the data collection process.

2. In "Results and Table 3", please describe briefly how often were the occurrences of sleep deprivation or poor sleep quality due to internet addiction (1-2 times a week, daily etc.?) Was this measured in the tests?

- Thank you for your comment. In this regards the sleep deprivation and poor sleep quality were assessed as a part of Pittsburgh Sleep Quality Index, while the internet addiction was measured through Young’s Internet Addiction Test. The value of poor sleep quality vs. internet addition is based on cross-tabulation. Due to being a cross-sectional study, we cannot specify how often the occurrences of sleep deprivation is due to internet addiction, and we can only reflect what is the distribution of different attributes of sleep deprivation across internet addition.

3. Were the poor sleep quality individuals taking any naps during the day and was this affecting their overall performance in the school exams?

- Thank you. While daytime dysfunction was assessed, specific data regarding daytime napping and its impact on academic performance were not collected. We have added this as a recommendation for future research in the limitations section.

4. In "Results and Table 4", was exercise or any physical activity (riding a bicycle to school) recorded among the participants and take into consideration with the survey results? Please describe briefly.

- No, we have not measured the attributes related to physical activity in our study. Our study area Dakshinkali Municipality lies in the mountainous regions where taking bicycle to school is not common as compared to Terai regions of Nepal, so it never occurred to us. In regards to other physical activities such as school games, daily exercise and other, initially we had planned to explore these factors but considering the volume and nature of questionnaire (self-administered) and complexity to properly report physical activity in terms of their frequency, intensity, duration and diverse nature of activity, we did not assess it as we were more focused on internet addition and sleep. We have addressed this in the limitation section. Thank you.

5. In "Results and Table 5", did the tests include questions that could inform if the poor sleep quality and internet usage among the adolescents in the study was higher during the weekends or holiday period compared to weekdays? Please describe briefly.

- No, we did not specifically explore variations in internet usage and sleep quality across weekends or holidays. Recognizing the importance of these factors, we have recommended their inclusion in future research. Thank you.
